# Quantitative analysis of the effect of ocular rotation on postoperative residual astigmatism in small incision lenticule extraction for myopia correction

**DOI:** 10.3389/fcell.2025.1695775

**Published:** 2025-12-04

**Authors:** Caiyu Liu, Xiaoying Xu, Hao Gu, Chi Wang, Lu Lu, Kaizhen Ye, Yan Zheng, Haiyan Wang, Wei Chen, Meiyan Li, Xiaolin Zhou, Shangkun Ou, Fangwen Yang

**Affiliations:** 1 Department of Ophthalmology, Second Affiliated Hospital of Guizhou Medical University, Kaili, China; 2 School of Clinical Medicine, Guizhou Medical University, Guiyang, China; 3 Department of Ophthalmology, Affiliated Hospital of Guizhou Medical University, Guiyang, China; 4 Department of Ophthalmology, Key Lab of Myopia, Eye & ENT Hospital of Fudan University, Ministry of Health, Shanghai, China; 5 Department of Ophthalmology, The Qinglong County People’s Hospital, Qinglong, Guizhou, China

**Keywords:** small incision lenticule extraction (SMILE), ocular rotation, astigmatism, vector analysis, refractive surgery

## Abstract

**Objective:**

The aim of this study was to conduct a systematic investigation into the effects of ocular rotation on postoperative residual astigmatism in patients undergoing small incision lenticule extraction (SMILE).

**Methods:**

A prospective observational cohort study involved 79 patients (153 eyes) with myopia and astigmatism who underwent SMILE surgery. Ocular rotational magnitude was measured using manual corneal and scleral marking with a slit-lamp microscopy assessment. Preoperative and postoperative (1- and 3-month) assessments included uncorrected distance visual acuity (UDVA), best-corrected visual acuity (BCVA), refractive error, and other relevant ocular parameters.

**Results:**

Residual astigmatism showed significant correlations with ocular rotation magnitude (r = 0.429, *p* < 0.001), preoperative intraocular pressure (r = −0.178, *p* = 0.032), and preoperative cylindrical lens power (r = 0.175, *p* = 0.035). A multiple linear regression analysis indicated that rotation magnitude significantly impacted postoperative residual astigmatism (*p* < 0.001). However, preoperative intraocular pressure (*p* = 0.349) and spherical equivalent (*p* = 0.105) were not significantly related to residual astigmatism. Linear regression analysis further demonstrated significant positive correlations between rotation amplitude and various astigmatism parameters at both 1- and 3-month postoperative follow-ups (all *p* < 0.05). In particular, the relationships were quantified as follows: cylindrical lens (CYL [D]) (1 month: y = 7.058x + 17.480, *p* < 0.001; 3 months: y = 7.464x + 13.610, *p* < 0.001), target-induced astigmatism (TIA [D]) (1 month: y = 0.112x + 1.275, *p* = 0.012; 3 months: y = 0.097x + 1.217, *p* = 0.026), surgically induced astigmatism (SIA [D]) (1 month: y = 0.094x + 0.936, *p* < 0.001; 3 months: y = 0.059x + 0.911, *p* = 0.022), and difference vector (DV [D]) (1 month: y = 0.041x + 0.289, *p* = 0.005; 3 months: y = 0.037x + 0.866, *p* = 0.011). Notably, rotation amplitude exhibited the strongest association with postoperative CYL. Receiver operating characteristic (ROC) analysis determined the optimal thresholds for rotation magnitude in predicting residual astigmatism to be 1.5° at 1 month (AUC = 0.753; sensitivity 79.7%; specificity 58.2%) and 2.5° at 3 months (AUC = 0.929; sensitivity 92.9%; specificity 83.5%).

**Conclusion:**

The magnitude of rotation shows a notably positive correlation with residual astigmatism during both the 1- and 3-month postoperative follow-ups. Thresholds of 1.5° (1 month) or 2.5° (3 months) prove predictive of residual astigmatism, with enhanced diagnostic precision at the later follow-up.

## Introduction

Femtosecond laser small incision lenticule extraction (SMILE) is a minimally invasive, flapless refractive surgical technique that uses femtosecond laser technology (Liu, 2022). In this procedure, the micro-lens is extracted via a peripheral corneal incision of approximately 2–3 mm. SMILE offers multiple benefits, such as improved biomechanical stability, reduced damage to the sub-basal nerve plexus, and a lower incidence of dry eye symptoms (Huang et al., 2024; Sharma et al., 2022). As such, it has emerged as a leading corneal refractive surgery for correcting myopia and astigmatism (Cui et al., 2023). Although SMILE exhibits outstanding efficacy in correcting myopia (Qian et al., 2020), its performance in addressing astigmatism is still not optimal (Song et al., 2023). Key factors that impact astigmatic results encompass the precision of surgical parameters, variations in ocular anatomy among individuals (astigmatic types and magnitudes), and the management of ocular rotation—each of these has the potential to undermine correction accuracy and diminish post-surgical visual quality (Yu et al., 2025; Xu et al., 2024; Moshirfar et al., 2020).

A significant technical hurdle is posed by positional ocular rotation, which is primarily driven by vestibular reflexes as patients shift from a seated to a supine position, and is called static ocular rotation (Pajic et al., 2017). This rotational displacement can cause misalignment between the intended astigmatic axis and the actual laser application, which may undermine the programmed ablation parameters and lead to an inadequate correction of both the magnitude and axis of astigmatism (Köse, 2020; Chen et al., 2019; Wei et al., 2024). Although intraoperative compensatory techniques are commonly used to address rotational errors and enhance surgical precision, the present VisuMax femtosecond laser platform does not feature integrated dynamic cyclotorsion tracking (Yang et al., 2024). This technological limitation could significantly impact surgical outcomes, particularly in cases of moderate-to-high astigmatism (Sachdev et al., 2023). Although SMILE has proven to be safe and effective in correcting myopic astigmatism, postoperative issues such as under-correction and regression remain, particularly in patients with high preoperative astigmatism (Reinstein et al., 2022; Teo and Ang, 2024).

At present, the AI-based method for measuring cyclotorsion—which relies on segmenting the optic disc and fovea in fundus images—proves difficult to adapt to the SMILE surgical setting (Wan et al., 2025; Zheng et al., 2022). Given these considerations, our study uses intraoperative corneal and scleral marking, along with postoperative slit-lamp observation, to quantify static ocular rotational parameters during surgery. Additionally, we utilize the Alpins vector analysis method to examine astigmatism vectors. This allows us to investigate the relationship between position-induced ocular rotation during SMILE surgery and postoperative residual astigmatism. Through this systematic approach, we establish a predictive model for surgical outcomes, further refining clinical strategies for personalized laser ablation protocols. Our results offer valuable insights for optimizing visual quality by enhancing preoperative planning and developing real-time rotational compensation algorithms.

## Study design and methodology

### Object

**FIGURE 1:**
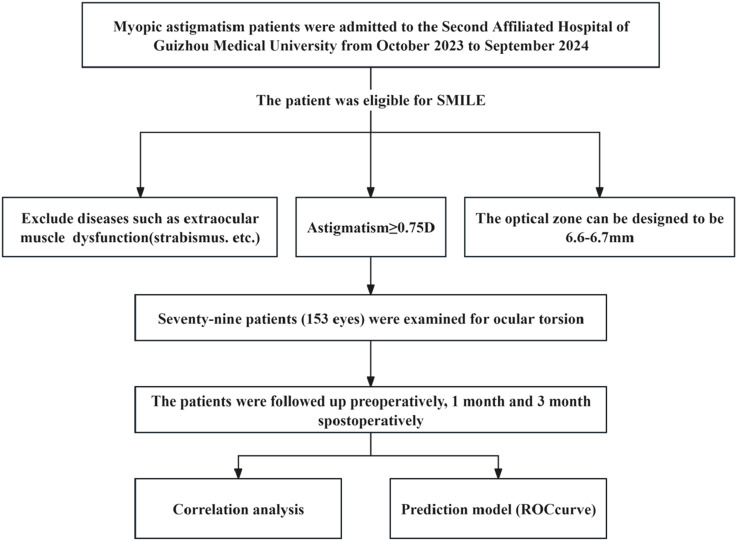
Research flowchart.

This prospective observational study was conducted at the Second Affiliated Hospital of Guizhou Medical University from October 2023 through September 2024. Eligible participants met standard SMILE surgery indications while presenting with astigmatism ≥0.75 diopters. Surgical parameters included an optical zone design of 6.6–6.7 mm. Exclusion criteria comprised ocular comorbidities, including extraocular muscle dysfunction (Figure 1).

## Methods

### Surgical and marking procedures

All surgeries were performed by a single experienced surgeon using the VisuMax femtosecond laser system (Carl Zeiss Meditec AG; 500 kHz). Laser parameters included an optical zone diameter ranging from 6.6 to 6.7 mm, a corneal cap diameter of 7.7 mm, a corneal cap thickness of 120 μm, an incision position at 90°, an incision length of 2 mm, and the corneal apex as the positioning center.

### Ocular rotation measurement methodology

**FIGURE 2:**
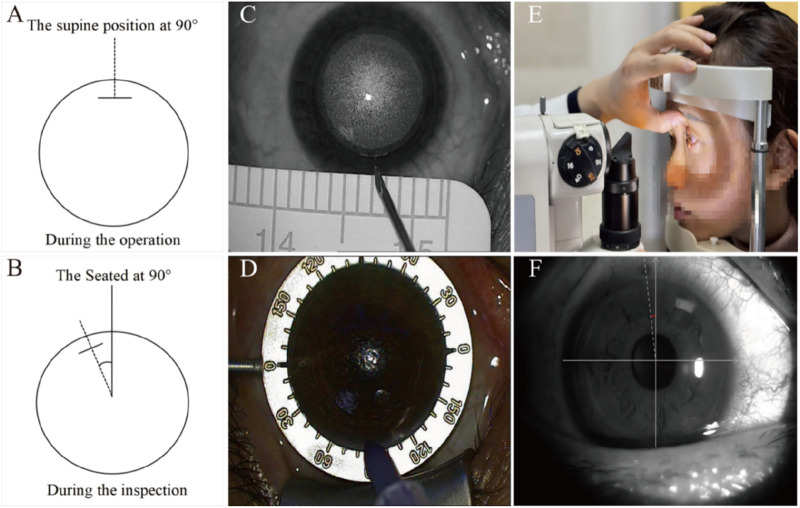
Diagram of ocular rotation and marking measurement. **(A)** Schematic diagram demonstrating ocular orientation at 90° in the supine position. **(B)** Schematic diagram demonstrating ocular orientation at 90° in the seated position. **(C)** Schematic representation of localization markers. **(D)** Schematic representation of chromogenic development and exposure demarcation. **(E)** Diaphragm light measurement diagram. **(F)** Slit-lamp microscopic examination images.

The design of the static ocular rotation measurement system is illustrated in Figures 2A,B. Accurate simulation of the spatial relationship between sitting (examination) and supine (surgical) eye positions is critical for both intraoperative marking and postoperative measurement.

### Key steps for intraoperative marking

All the markings and measurements were carried out by a single experienced doctor following a standardized procedure.

1. Following laser scanning (supine position), a sterile syringe needle (Figure 2C) was used to place a reference mark at the incision center.
2. After lens removal, the marked points were highlighted using a sterile surgical marker (Figure 2D).
3. Immediately postoperatively, the rotational amplitude was measured and recorded using slit-lamp microscopy (sitting position, Figure 2E).

### Slit-lamp measurement protocol

1. The marked position was identified under broad illumination, after which the slit beam was narrowed to its finest setting (Figure 2F).
2. The beam was aligned parallel to the marked incision.
3. Rotational magnitude was recorded directly from the slit lamp’s angular scale.

### Preoperative and postoperative evaluation parameters

Comprehensive ophthalmic examinations were carried out before surgery, along with at 1- and 3-month intervals following the procedure. The standardized assessments encompassed uncorrected distance visual acuity (UDVA), best-corrected visual acuity (BCVA), autorefraction and subjective refraction (including sphere, cylinder, and axis measurements), non-contact tonometry for intraocular pressure (IOP) readings, and slit-lamp biomicroscopy for anterior segment evaluation. For consistency, a senior ophthalmic technician conducted all measurements under uniform conditions.

### Vector analysis method for astigmatism

The Alpins vector analysis method is used to evaluate the effectiveness of astigmatism correction, focusing on key parameters such as magnitude and axial direction. These parameters include the following:

1. Target-induced astigmatism (TIA), which represents the intended surgical correction magnitude and corresponds to preoperative astigmatism.
2. Surgically induced astigmatism (SIA), reflecting the actual astigmatic correction achieved through surgery.
3. Difference vector (DV), indicating the residual astigmatism that is the vector difference between SIA and TIA.
4. Angle of error (AoE), which shows the angular difference between SIA and TIA vectors. An AoE of 0 signifies perfect alignment, while a positive AoE denotes counterclockwise SIA rotation, and a negative AoE indicates clockwise SIA rotation.
5. Measurement error (ME), representing the magnitude of discrepancy between TIA and SIA.
6. Correction index (CI), the ratio of SIA to TIA. A CI of 1 signifies complete correction, less than 1 indicates undercorrection, and greater than 1 suggests overcorrection.
7. Index of success (IOS) is the ratio of DV to TIA, where an IOS of 0 represents perfect correction (Supplementary Figure S1).

### Statistical analysis

All statistical analyses were conducted using SPSS 27.0 (IBM Corp), with additional data organization carried out in Excel (Microsoft). Continuous variables were presented as the mean ± SD for data following normal distribution or as the median (interquartile range) for non-normally distributed data, as ascertained using the Kolmogorov–Smirnov test. Categorical variables were summarized as frequencies (percentages). The analysis of astigmatism vectors relied on the Alpins method. Correlation analyses involved the use of Pearson coefficients for normally distributed continuous variables and Kendall coefficients for ordinal or nonparametric data. To assess potential factors associated with postoperative residual astigmatism, multiple linear regression analysis was used. Furthermore, the predictive value of rotation magnitude for postoperative residual astigmatism was examined using receiver operating characteristic (ROC) curve analysis. Statistical significance was established at *p* < 0.05 (two-tailed).

## Results

### Demographic and clinical characteristics

The study cohort consisted of 79 patients (153 eyes) with myopic astigmatism who underwent SMILE surgery. Table 1 presents their descriptive characteristics. In terms of rotation incidence, 82.35% of eyes (126/153) exhibited rotation, while 17.65% (27/153) maintained a stable position without any rotation. Regarding rotation magnitude, the mean was 2.44° ± 1.93°, with a range of 1°–11° (Table 1). It is important to note that all procedures effectively corrected both myopia and astigmatism, and there were no intraoperative or postoperative complications.

**TABLE 1:** Preoperative general information.

Parameter	n
Gender: male Female	48 (60.8%) 31 (39.2%)
Eye signal: right Left	78 (50.8%) 75 (49.2%)
Age	22.92 ± 6.01
Mmagnitude of ocular rotation	2.44 ± 1.93 (0°–11°)
No rotate Rotate	27 (17.65%) 126 (82.35%)
Optical zone (mm)	6.67 ± 0.11
Axial length of the eye (mm)	25.65 ± 0.92
Kappa (x, y)	(−0.04 ± 0.11, −0.06 ± 0.12)
WTW (mm)	11.66 ± 0.41
UDVA (logMAR)	3.94 ± 0.34
IOP (mmHg)	16.47 ± 2.71
Diopter (D)	
Spherical lens	−4.26 ± 1.46
Cylindrical lens	−110.27 ± 69.71
Axial	108.87 ± 73.29

WTW, white to white; UDVA, uncorrected distance visual acuity; IOP, intraocular pressure.

### Vector analysis of astigmatism

Preoperative analysis showed that the average TIA in our study group was 0.99 D, with a vector mean of 0.75 D at 1.6°, X = 0.81, and Y = 0.37 (Supplementary Figure S2A). Postoperatively, the average residual astigmatism decreased to 0.30 D, with a vector mean of 0.10 D at 19.3°, X = 0.29, and Y = 0.25 (Supplementary Figure S2B). The average SIA was found to be 1.03 D, with a vector mean of 0.66 D at 179.0°, X = 0.87, and Y = 0.48 (Supplementary Figure S2C). The observed difference between the TIA and SIA vectors points to quantifiable inaccuracies in our astigmatism correction approach. Importantly, the average residual astigmatism of 0.30 D indicates a systematic trend toward undercorrection (Supplementary Figure S2).

### Correlation analysis of postoperative astigmatism

Pearson correlation analysis demonstrated notable relationships between postoperative astigmatism and three specific parameters (all *p* < 0.05, Table 2): rotation magnitude (r = 0.429, *p* < 0.001, Table 2), preoperative intraocular pressure (r = −0.178, *p* = 0.032, Table 2), and preoperative cylindrical lens power (r = 0.175, *p* = 0.035, Table 2). Among these, the strongest correlation emerged with rotation magnitude (r = 0.429, *p* < 0.001, Table 2). None of the other evaluated parameters exhibited significant correlations (all *p* > 0.05, Table 2).

**TABLE 2:** Correlation analysis of postoperative astigmatism.

Indicator	Average ± standard deviation	Correlation coefficient	*p*-value
Gender (male = 1, female = 0)	0.640 ± 0.481	−0.089	0.290
Age	22.94 ± 6.018	0.028	0.737
Eye signal (right = 1, left = 0)	0.510 ± 0.502	0.095	0.255
Magnitude of ocular rotation	2.910 ± 2.366	0.429	<0.001***
Optical zone (mm)	6.671 ± 0.115	−0.019	0.819
AL (mm)	25.656 ± 0.917	−0.107	0.201
IOP (mmHg)	16.470 ± 2.707	−0.178	0.032*
Kappa (X)	−0.043 ± 0.112	−0.070	0.401
Kappa (Y)	0.061 ± 0.117	0.035	0.674
WTW (mm)	11.665 ± 0.411	−0.109	0.194
Preoperative cylinder lens (D)	−110.27 ± 69.71	0.175	0.035*

*, *p* < 0.05; ***, *P* < 0.001; AL, axial length; IOP, intraocular pressure; WTW, white to white.

### Multivariate linear regression analysis

Based on our correlation findings presented in Table 2, we conducted a multiple linear regression analysis to assess the factors that might forecast postoperative residual astigmatism. We considered rotation magnitude, preoperative intraocular pressure (IOP), and preoperative spherical equivalent as potential predictors. As outlined in Table 3, our analysis revealed a highly significant correlation between rotation magnitude and residual astigmatism (*β* = 0.609, *p* < 0.001, Table 3). However, preoperative IOP (*β* = 0.062, *p* = 0.349, Table 3) and spherical equivalent (*β* = −0.106, *p* = 0.105, Table 3) did not exhibit significant predictive power in the final regression model.

**TABLE 3:** Gradual regression of residual astigmatism after surgery.

Indicator	*β*	*t*	*p*-value	*R* (Huang et al., 2024)
Magnitude of ocular rotation (°)	0.609	9.298	<0.001***	
IOP (mmHg)	0.062	0.940	0.349	0.393
Preoperative cylinder lens (D)	−0.106	−1.633	0.105	

Dependent variable, residual astigmatism after surgery; *β*, regression coefficient; *t*, significance; *R* ^2^, coefficient of determination; *p < 0.05, **p < 0.01, and ***p < 0.001 indicate statistical significance; IOP, intraocular pressure.

### Correlation analysis of rotational magnitude and postoperative astigmatism vector

Pearson correlation analysis revealed notable relationships between rotation magnitude and postoperative refractive outcomes during the 1- and 3-month follow-ups (Table 4). The strongest correlation was observed between rotation magnitude and cylinder power (CYL), exhibiting substantial correlation coefficients at both the 1-month (r = 0.521, *p* < 0.001, Table 4) and 3-month (r = 0.768, *p* < 0.001, Table 4) evaluations. Although significant, lesser correlations were found with SIA [D] (1-month: r = 0.269, *p* < 0.001; 3-month: r = 0.185, *p* = 0.022, Table 4), TIA [D] (1-month: r = 0.202, *p* = 0.012; 3-month: r = 0.180, *p* = 0.026, Table 4), and DV [D] (1-month: r = 0.228, *p* = 0.005; 3-month: r = 0.214, *p* = 0.011, Table 4).

**TABLE 4:** Correlation analysis of rotation magnitude and astigmatism vector (1 and 3 months after surgery).

Indicator	One month after the operation			Three months after the operation		
	Astigmatism (*X ± S*)	*r*	*p*	Astigmatism (*X ± S,*)	*r*	*p*
Postoperative CYL	−0.387 ± 0.291	0.521	<0.001*	−0.304 ± 0.258	0.768	<0.001*
Postoperative AXIS	78.100 ± 53.622	0.026	0.770	77.970 ± 57.954	0.046	0.631
SIA (D)	1.167 ± 0.679	0.269	<0.001*	1.056 ± 0.622	0.185	0.022*
SIA (°)	30.519 ± 39.463	−0.030	0.712	26.954 ± 41.029	−0.017	0.832
TIA (D)	1.549 ± 1.072	0.202	0.012*	1.455 ± 1.041	0.180	0.026*
TIA (°)	−7.899 ± 37.898	−0.095	0.241	−10.622 ± 39.369	−0.083	0.309
AE (°)	96.180 ± 50.524	−0.010	0.899	92.040 ± 60.037	−0.021	0.795
ME (D)	0.382 ± 0.630	0.055	0.501	0.399 ± 0.615	0.119	0.114
DV (D)	0.389 ± 0.346	0.228	0.005**	0.953 ± 0.334	0.214	0.011*
DV (°)	−25.732 ± 89.981	−0.127	0.117	−26.157 ± 83.943	−0.126	0.122
IOS	0.358 ± 0.407	0.027	0.742	1.206 ± 3.013	0.097	0.254
CI	0.955 ± 0.784	−0.070	0.392	1.023 ± 1.704	−0.118	0.145

SIA, surgically induced astigmatism (magnitude [D] and axis [°]); TIA, target-induced astigmatism (magnitude [D] and axis [°]); AE, angle of error (°); ME, magnitude of error (D); DV, difference vector (magnitude [D] and axis [°]); IOS, index of success; CI, correction index; statistical significance: *p < 0.05, **p < 0.01, and ***p < 0.001.

### Linear correlation analysis

**FIGURE 3:**
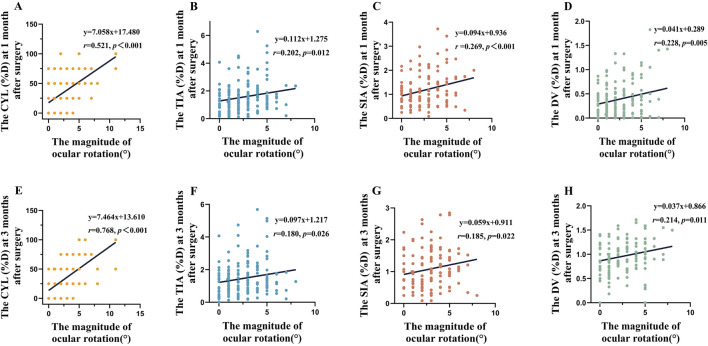
The magnitude of rotation demonstrates a linear relationship with CYL, TIA, SIA and DV. **(A)**, CYL(D) at 1-month postoperative follow-up. **(B)**, TIA(D) at 1-month postoperative follow-up. **(C)**, SIA(D) at 1-month postoperative follow-up. **(D)**, DV(D) at 1-month postoperative follow-up. **(E)**, CYL(D) at 3-month postoperative follow-up. **(F)**, TIA(D) at 3-month postoperative follow-up. **(G)**, SIA(D) at 3-month postoperative follow-up. **(H)**, DV(D) at 1-month postoperative follow-up.

Linear regression analysis demonstrated a notably positive correlation between the magnitude of rotation and postoperative CYL (1-month: y = 7.058x + 17.480, *p* < 0.001; 3-month: y = 7.464x + 13.610, *p* < 0.001, Figures 3A,E), TIA (1-month: y = 0.112x + 1.275, *p* = 0.012; 3-month: y = 0.097x + 1.217, *p* = 0.026, Figures 3B,F), SIA (1-month: y = 0.094x + 0.936, p*P* < 0.001; 3-month: y = 0.059x + 0.911, *p* = 0.022, Figures 3C,G), and DV (1-month: y = 0.041x + 0.289, *p* = 0.005; 3-month: y = 0.037x + 0.866, *p* = 0.011, Figures 3D,H) at both 1- and 3-month follow-ups.

### Predictive value of rotational magnitude for residual astigmatism

**FIGURE 4:**
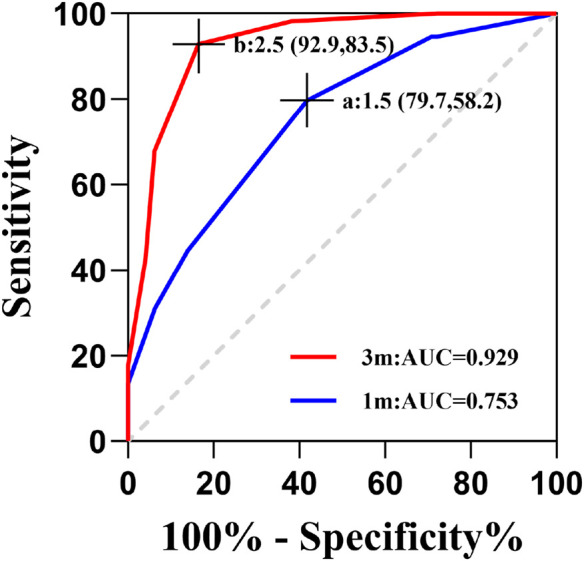
Predictive performance of ocular rotation for postoperative residual astigmatism. **(A)** A rotational truncation cutoff of 1.5° at 1 month postoperatively achieved 79.7% sensitivity and 58.2% specificity; **(B)** a rotational truncation cutoff of 2.5° at 3 months postoperatively achieved 92.9% sensitivity and 83.5% specificity.

The ROC curve analysis revealed notable temporal variation in the predictive capability of rotational magnitude regarding postoperative residual astigmatism, as illustrated in Figure 4. In particular, at 3 months after surgery, the rotational magnitude exhibited remarkable diagnostic precision (AUC = 0.929). Its optimal predictive performance was achieved at a threshold of 2.5°, yielding a sensitivity of 92.2% and a specificity of 83.5%. However, the predictive value at 1 month postoperatively was considerably lower (AUC = 0.753), with the best cutoff point being 1.5°, corresponding to a sensitivity of 79.7% and a specificity of 58.2%.

## Discussion

In refractive surgery, the correction of astigmatism holds equal significance to the management of spherical equivalent (SE). Astigmatism, as a vector quantity comprising both magnitude and axis components, requires careful surgical planning that considers both these parameters to achieve optimal visual results (Cui et al., 2023). Although SMILE has proven effective in addressing high myopic astigmatism, repeated comparative analyses indicate that its astigmatic correction is not as optimal as that achieved by LASIK, because in LASIK, the integrated iris registration system automatically corrects for static cyclotorsion by detecting unique iris features, thereby ensuring precise astigmatic alignment (Song et al., 2023; Hou et al., 2024; Jiao et al., 2025). Specifically, cases involving high preoperative astigmatism (>2.00 D) often exhibit undercorrection and regression (Dishler et al., 2020). Multiple factors contribute to the variability in astigmatic correction achieved through SMILE surgery, such as preoperative astigmatism characteristics, centration accuracy, optical zone decentration, Kappa angle, and, as emphasized in our study, ocular rotation (Yujia, 2023). Among various factors, Chow et al. (2019) highlighted ocular rotation as a key factor influencing astigmatic outcomes, a conclusion reinforced by our quantitative vector analysis. Ocular rotation involves two distinct components. Dynamic rotation, occurring intraoperatively, is often caused by inadequate fixation but is effectively mitigated by the suction ring, minimizing its clinical significance (Yang et al., 2023). Static rotation, on the other hand, is a consequence of physiological vestibulo-ocular reflexes triggered by positional shifts (e.g., from sitting to supine) (Zhifeng et al., 2023; Hua et al., 2017). The present work specifically examines cyclotorsion resulting from postural changes and its implications for astigmatic correction in SMILE surgery. This form of cyclotorsion is mechanistically distinct from the postoperative rotational stability of a toric IOL as the former is primarily a physiological phenomenon (e.g., mediated by vestibulo-ocular reflexes), whereas the latter is influenced by capsular bag biomechanics, thus involving separate anatomical structures and rotational drivers (Kim and Chung, 2025). This study presents a new quantification method for ocular rotation amplitude that integrates corneal–scleral landmarking with slit-lamp measurement techniques. To the best of our knowledge, this is the first instance where precise rotational assessment, coupled with Alpins vector analysis, has been used to systematically assess the correlation between postural eye movement and postoperative residual astigmatism (Wang et al., 2025). The establishment of this quantitative model represents our central finding and provides a novel, evidence-based framework for personalizing rotational compensation in SMILE surgery. The dual-phase marking protocol, implemented both prior to and following lens removal, was devised to accomplish three key goals: first, to minimize dye loss during microscopic manipulation; second, to prevent stromal penetration by the marking dye; and third, to ensure accurate anatomical alignment even with surgical incision enlargement.

The ocular rotation during refractive surgery can cause significant deviations in the initial positioning of the laser cutting instrument, thereby substantially affecting astigmatic correction. Previous research indicates that uncompensated ocular rotations exceeding 2° during SMILE surgery may lead to undercorrection of astigmatism, with rotations of ≥4° resulting in approximately 14% undercorrection (Alio et al., 2013). Additionally, increasing degrees of rotation exacerbate both axial misalignment and the magnitude of undercorrection, ultimately impacting postoperative visual quality (Xu et al., 2025; Lee et al., 2025). Our data demonstrate that 85.7% of patients exhibited clinically significant static rotation (vs. 96% in a prior report), with magnitudes ranging 0°–11° (literature range: 0°–17°) (Ganesh et al., 2017; Zhang et al., 2021). This rotational shift results in measurable deviations from the intended ablation zones, thereby directly affecting vector accuracy. More precisely, our observations revealed a strong correlation between the magnitude of rotation and residual astigmatism (r = 0.429, *p* < 0.001). The peak predictive value was noted at 3 months postoperatively (AUC = 0.929, with a cut-off of 2.5°). Additionally, there was a significant trend of undercorrection (mean residual astigmatism = 0.30 D). Our findings further suggest a positive association between the degree of ocular rotation and residual astigmatism after surgery (postoperative 1 month: y = 7.058x + 17.480, r = 0.521, *p* < 0.001; postoperative 3 months: y = 7.464x + 13.630, r = 0.768, *p* < 0.001), which is consistent with previous studies. In clinical settings, ocular rotation is typically compensated for using scientific methods to ensure accurate laser ablation (Zhou et al., 2022). In excimer laser surgery, in particular, the combination of iris recognition and wavefront-guided technology proves effective in minimizing astigmatism and reducing axis misalignment (Razmjou et al., 2022). A comparative analysis of artificial limbal marking and iris positioning demonstrates that, in the absence of an automated system, artificial limbal marking serves as a reliable alternative for intraoperative adjustment of ocular rotation (Wang et al., 2022). Based on our findings, intraoperative rotational compensation is necessary for patients with a cyclotorsion magnitude exceeding 2.5°. However, since the VisuMax 500 platform lacks an automatic compensation mechanism, this adjustment must be performed manually. Although the latest VisuMax 800 system incorporates OcuLign eye rotation compensation technology, its implementation still relies on preoperative corneal marking and manual intraoperative correction (Ganesh et al., 2025). In essence, this technology does not fundamentally overcome the limitation of femtosecond laser systems in addressing cyclotorsion as it still relies on manual intervention. Therefore, within the current SMILE surgery, rotational compensation can only be achieved manually. Our findings indicate an urgent need for the development and widespread adoption of integrated, automated compensation systems in future platform iterations to optimize astigmatic outcomes. Our study provides a potential explanation for the suboptimal astigmatic outcomes in patients with high astigmatism (>2.00 D) following SMILE. We identified a dose-dependent relationship between uncompensated intraoperative cyclotorsion and postoperative residual astigmatism. This finding implies that even a technically perfect SMILE procedure may be compromised by undetected rotational misalignment, particularly in eyes with higher cylindrical errors. We suggest that a standardized preoperative workup should be incorporated, mandating the assessment of ocular cyclotorsion in both sitting and supine positions where necessary, with careful consideration of astigmatic severity. In particular, for patients with preoperative astigmatism greater than 2.00 D, it is recommended to thoroughly communicate the necessity of a personalized surgical plan. This should involve preoperatively measuring the degree of ocular cyclotorsion and, based on its magnitude, determining whether preoperative corneal marking and subsequent intraoperative rotational compensation are required to achieve superior postoperative outcomes.

This study supports the hypothesis that SMILE outcomes are significantly impacted by ocular rotation effects, mainly due to the absence of active rotational tracking in the VisuMax 500 femtosecond laser platform (Kanellopoulos, 2017). The current limitations of the system, particularly the lack of automated rotational displacement recognition and compensation, might lead to less-than-optimal correction of moderate-to-high astigmatism (Chang et al., 2022). These results echo previous findings by Dishler et al, who documented higher undercorrection rates in treatments for higher astigmatism. Importantly, our study revealed changes in the cutoff values for rotation size over time (1.5° at 1-month postoperative and 2.5° at 3-month postoperative), indicating that early postoperative corneal edema or ablation zone surface irregularities might partially mask rotational inaccuracies (Su et al., 2025). As tissue remodeling progresses, the true influence of rotation becomes more apparent. Consequently, longer follow-up durations allow for a more accurate assessment of the true effect of rotational error on astigmatic outcomes. For this reason, the 3-month results are accorded greater emphasis in our analysis, underscoring the need for future studies with extended follow-up periods. Our results highlight the need for artificial corneal marking to reduce ocular rotation during surgeries without automated tracking, particularly in SMILE procedures. However, this marking method relies on the surgeon’s skill and may lead to measurement inaccuracies, possibly explaining the moderately low sensitivity (58.2%) observed at the 1-month postoperative threshold. Future improvements, such as the integration of intraoperative real-time imaging, including OCT guidance or AI-assisted positioning, are expected to greatly improve compensation accuracy.

Several methodological constraints require careful attention in interpreting our findings. First, the inclusion of high astigmatism cases (cylindrical ≥2.00 D) accounted for only 12.4% of the sample. Although the relatively small proportion of high astigmatism cases may have influenced our findings, it is important to note that visual quality is often compromised in a majority of the population even with astigmatism exceeding 0.75 D. Therefore, although further studies with more high-astigmatism cases are needed to verify the generalizability of our results, the practical conclusions drawn from this study remain valid. Second, inherent measurement inaccuracies in the slit-lamp marking method may have led to an underestimation of the association between rotation amplitude and residual astigmatism. Third, crucial confounding factors such as dynamic rotation and ablation center offset were not considered in the analysis, and there are common factors such as the relatively short follow-up period and the small sample size. To overcome these limitations, future research should aim to achieve the following improvements: 1) expand the high-astigmatism cohort, extend follow-up periods, and conduct stratified analyses to clarify the dose–effect relationship of rotational effects; seek external validation through multi-center collaborations involving diverse patient populations and surgeons; 2) utilize advanced imaging techniques such as Scheimpflug imaging or three-dimensional corneal topography to improve the accuracy of rotation measurements; 3) incorporate objective scatter index (OSI) and wavefront aberration measurements (the inclusion of subjective (such as glare, halos, or other visual symptoms) metrics would have provided a more comprehensive assessment of postoperative quality of vision, and we consider this a meaningful direction for future investigation); and 4) develop personalized compensation algorithms based on vector analysis to enable coordinated correction of both rotation direction and amplitude. Although these limitations prevent definitive conclusions, the consistent dose–response relationships observed across various analytical approaches bolster the biological plausibility of our primary findings.

In summary, SMILE surgery exhibits impressive safety and efficacy in correcting myopic astigmatism. A key controllable factor influencing residual astigmatism is static eye rotation. Our study underscores the diagnostic value of eye rotation magnitude in anticipating postoperative residual astigmatism. This indicates that preoperative assessment of rotation parameters, coupled with refined intraoperative adjustment strategies, has the potential to improve astigmatism correction results. Furthermore, these insights facilitate an initial prognosis of the postoperative refractive state, particularly in patients with significant astigmatism and rotation amplitude of 2.5° or more, thus carrying considerable clinical importance. Forthcoming technological progress should focus on creating integrated, automated rotation-tracking systems to surpass present equipment constraints and attain greater refractive correction accuracy.

## Conclusion

This study demonstrates that the magnitude of ocular rotation plays a significant role in determining the outcomes of astigmatism correction. Our results highlight postural ocular rotation as a clinically adjustable factor that contributes to postoperative residual astigmatism. Through quantitative analysis, we observed notable positive linear relationships between rotational magnitude and residual cylinder power at both 1-month (r = 0.521, *p* < 0.001) and 3-month (r = 0.768, *p* < 0.001) postoperative time points. Clinically significant rotation thresholds were set at ≥1.5° at 1 month and ≥2.5° at 3 months, with the latter threshold proving more effective in predicting residual astigmatism (AUC = 0.929).

## Data Availability

The original contributions presented in the study are included in the article/Supplementary Material, further inquiries can be directed to the corresponding authors.
