# Supplementary material for: Quantitative analysis of the effect of ocular rotation on postoperative residual astigmatism in small incision lenticule extraction for myopia correction
Source: Front Cell Dev Biol. 2025 Dec 4;13:1695775. doi: 10.3389/fcell.2025.1695775 (PMC12713198; doi:10.3389/fcell.2025.1695775)
Supplement: Supplementary file 1 [file DataSheet1.docx]

**Supplementary** **Figures and** **Legends**

**
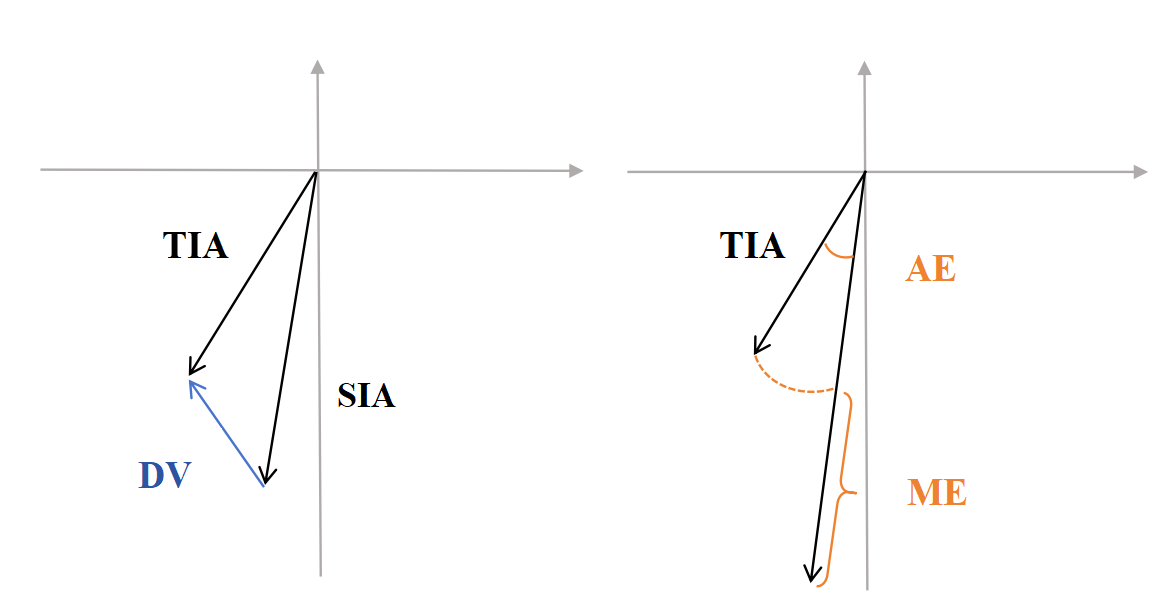
**

**S Fig.1 Vector Analysis Method for Astigmatism.**Note:SIA: Surgically induced astigmatism (magnitude [D] and axis [°]);TIA: Target induced astigmatism (magnitude [D] and axis [°]);AE: Angle of error (°);ME: Magnitude of error (D);DV: Difference vector (magnitude [D] and axis [°]).

**
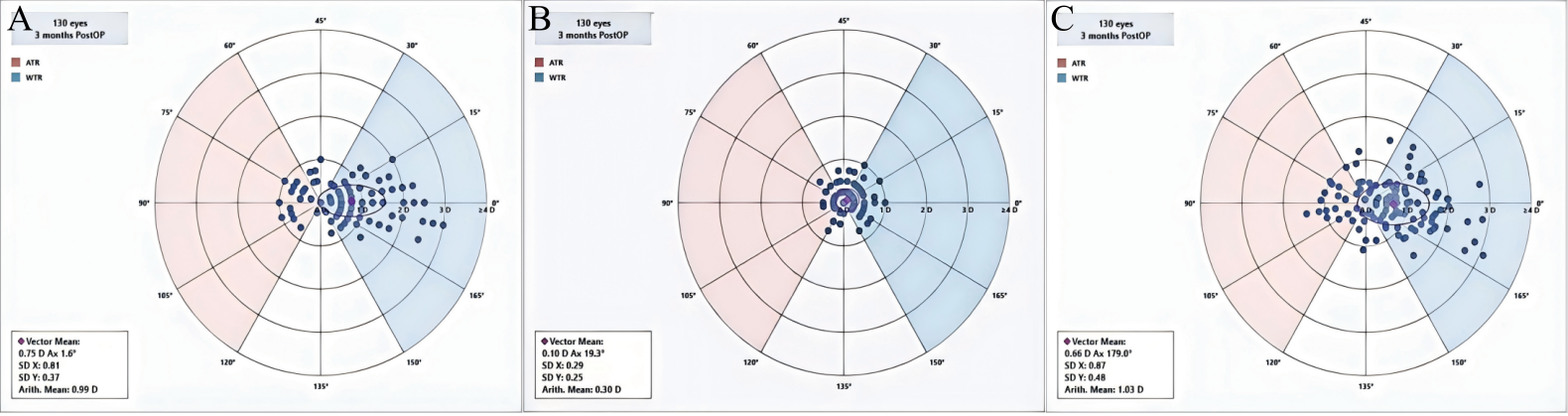
**

**S Fig.2 Vector Analysis of Astigmatism.** A, Preoperative astigmatism. B, Postoperative astigmatism. C, Surgically Induced Astigmatism(SIA).
